# Anterior Cruciate Ligament Repair: Surgical Technique Using Augmentation With Biocomposite Scaffold

**DOI:** 10.1002/atn2.70064

**Published:** 2026-05-05

**Authors:** Caleb Berta, Elizabeth Powell, Joshua Broussard, Thomas Evely, Walter Smith, Kevin Williams, Amit Momaya

**Affiliations:** ^1^ Department of Orthopaedic Surgery University of Alabama at Birmingham Birmingham Alabama U.S.A.

## Abstract

Repair of the anterior cruciate ligament has recently regained interest over the past decade. Such repairs can be augmented with nonabsorbable suture for strength or biologic scaffolds to promote healing. This technique uses the BioBrace, a porous 3‐dimensional collagen scaffold made from bioresorbable bovine type I collagen that is reinforced with poly(L‐lactic acid) microfilaments. The scaffold provides mechanical load‐sharing properties and can increase biologic healing potential. This technique describes an anterior cruciate ligament repair with BioBrace augmentation.

## Graphical abstract

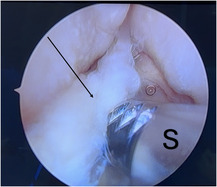

**VIDEO 1:** This technique shows anterior cruciate ligament (ACL) repair augmented with a biocomposite scaffold. The knee is positioned at 90° of flexion, and the borders of the patella are marked. Standard anteromedial and anterolateral portals are established. An arthroscope is introduced into the knee, followed by a shaver to debride the synovium and tissue surrounding the ACL tear. A notchplasty is then performed to widen the intercondylar notch and improve visualization. The ACL tear is identified near the proximal portion of the ligament, close to its posterolateral femoral insertion. While the surgeon inspects the knee, qualified personnel prepare the BioBrace by passing it through a knotless suture anchor and folding it over to double its tensile strength. A whipstitch is then performed 2 cm proximal to the distal end, extending to the most distal portion. A luggage tag suture is placed into the proximal portion of the ACL, where adequate tissue is present for strong fixation. The suture is tensioned, and a second luggage tag suture is similarly placed and tightened. A tibial guide is then used to drill a 2.4‐mm guidewire through the tibia, followed by drilling with a 3.5‐mm drill. A suture passer is used to insert a suture through the tunnel to maintain the tunnel for subsequent passage of the BioBrace. The luggage tag sutures and BioBrace are loaded into the knotless suture anchor. A drill and hand tap are used to prepare the femoral socket, and the knotless suture anchor (loaded with the BioBrace and sutures) is inserted into the femoral notch. Excess suture is trimmed. The BioBrace is then pulled down through the tibial tunnel and fixated using another knotless suture anchor. The repair is then assessed for appropriate tension and stability. Video content can be viewed at https://doi.org/10.1002/atn2.70064.

Anterior cruciate ligament (ACL) repair fell out of favor after earlier studies showed inferior outcomes when compared with ACL reconstruction.^1^ However, with new surgical techniques and the development of biological augmentation strategies, ACL repair has reemerged as an alternative option for patients with certain tear patterns.^2^, ^3^ Repair preserves the native stump's proprioceptive potential while decreasing complications associated with autograft harvest, such as hamstring or quadriceps weakness, risk of native tendon rupture, anterior knee pain, patellar fracture, and cramping.^4^, ^5^, ^6^ These advantages of ACL repair may lead to faster return of range of motion and a more natural‐feeling knee, as shown by higher forgotten knee scores (FJS‐12).^7^

The success of ACL repair can be attributed to stricter indications coupled with newer techniques that can assist in biological and mechanical augmentation, such as biodegradable collagen scaffolds and suture tape augmentation.^8^ The collagen scaffolds help promote healing of the torn ACL, while the suture tape augmentations provide an additional structure that acts as a load‐sharing construct to strengthen the ACLR.^9^, ^10^ The BioBrace (ConMed Linvatec, Largo, FL, USA) is a biologic porous bovine collagen scaffold that combines the mechanical load‐sharing properties of suture tape augmentation with the biologic healing potential of the biodegradable collagen scaffolding.^11^ In this paper, we describe surgical techniques, fixation methods, clinical indications, and advantages and disadvantages of ACL repair with BioBrace augmentation (Table 1).

**TABLE 1:** Advantages and Disadvantages of Repair With BioBrace

Advantages	Disadvantages
BioBrace provides both biological healing properties and enhanced mechanical support	The increased thickness of the construct can make tunnel passage more difficult
Provides support for approximately 2 years before naturally resorbing	Increased cost compared with traditional reinforcement methods
Compatible with knotless fixation systems	The technique may be unfamiliar and require additional preparation time
May reduce micromotion and support early rehabilitation due to augmented strength	Improper suture tensioning or folding can compromise tension
BioBrace material is visibly integrated into the repair site, so the surgeon can see the reinforced structure while constructing or tensioning the graft	Limited long‐term data on outcomes

## SURGICAL TECHNIQUE

Surgical techniques for graft preparation are presented in Video 1. The pearls and pitfalls with BioBrace are described in Table 2.

**TABLE 2:** Pearls and Pitfalls of Repair With BioBrace

Pearls	Pitfalls
When folded over to double the BioBrace, it increases the load‐sharing strength	The added bulk from doubling over the BioBrace can complicate graft passage. This may require whipstitching each end of the BioBrace separately and passing them individually
Placing the BioBrace alongside the native ACL fibers improves contact area and optimizes biologic integration	Improper alignment or twisting of the BioBrace may compromise graft orientation
A consistent tensioning technique helps ensure the BioBrace load‐shares with the native tissue rather than over‐tightening the repair	Failure to properly tension the BioBrace may result in insufficient augmentation of the repair
The BioBrace is compatible with most fixation systems, allowing integration into existing ACL repair workflows	Improper fixation may lead to slippage or inadequate capture of the BioBrace

ACL, anterior cruciate ligament.

### Knee Preparation

**FIGURE 1:**
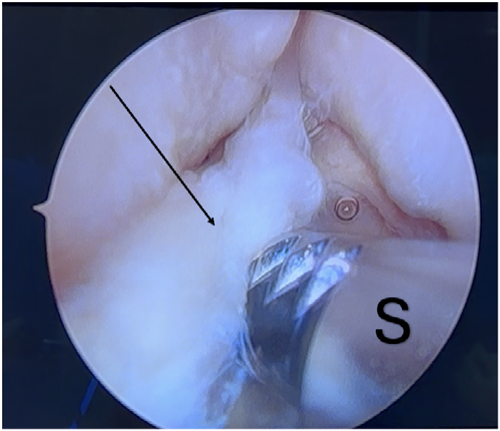
Shows a 30° arthroscopic view of the right knee. A shaver (S) has been introduced into the knee to debride excess tissue and synovium (arrow) surrounding the ACL tear. (ACL, anterior cruciate ligament.)

**FIGURE 2:**
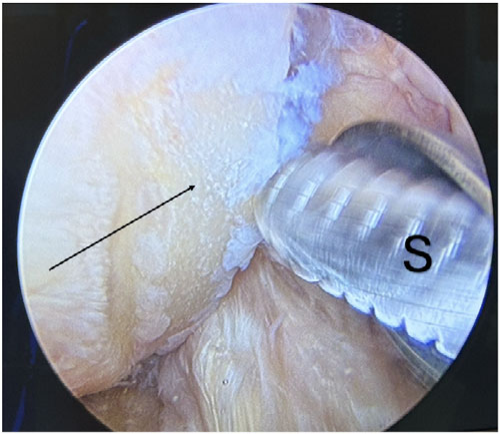
Shows a 30° arthroscopic view of the lateral aspect of the right knee. A notchplasty is performed using a shaver (S) to widen the intercondylar notch (arrow).

The knee is placed into 90° of flexion and is marked out around the border of the patellar tendon. Standard anteromedial and anterolateral portals are established. The arthroscope is introduced into the knee, followed by a shaver to debride excess tissue and synovium to evaluate the ACL tear and determine if the remaining stump is viable for repair (Figure 1). A notchplasty can be performed using a shaver to widen the intercondylar notch and improve visualization (Figure 2).

### BioBrace Preparation

**FIGURE 3:**
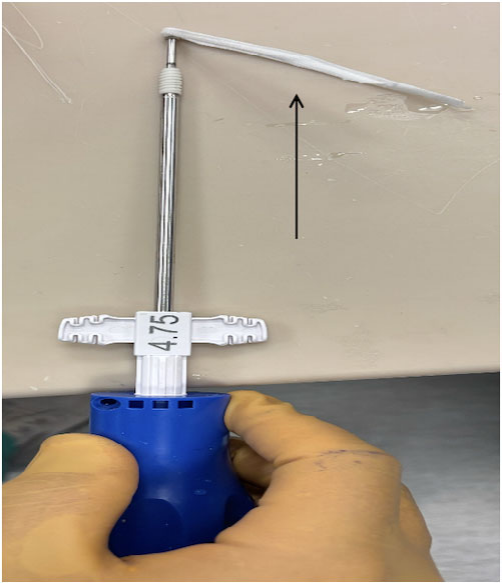
Shows a folded 100 mm BioBrace (arrow) looped through a 4.75‐mm knotless suture anchor.

**FIGURE 4:**
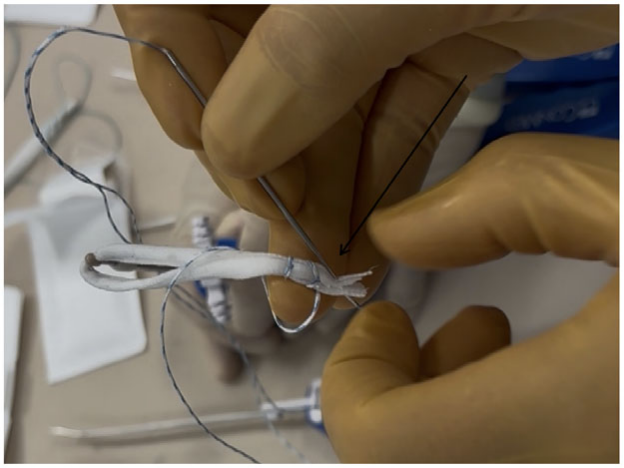
Shows whipstitching (arrow) of the folded BioBrace. Whipstitching starts approximately 2 cm from the open end and continues distal from the closed end.

**FIGURE 5:**
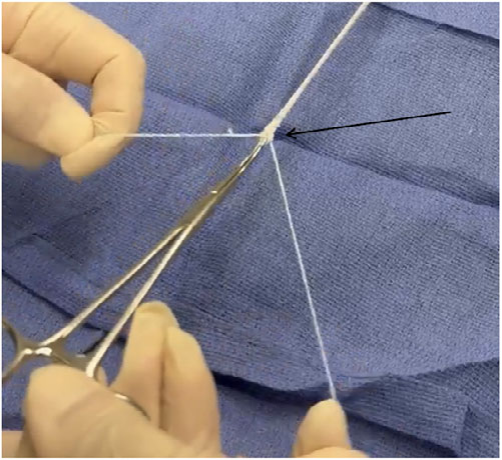
Shows whipstitching (arrow) of the unfolded end of the BioBrace. Whipstitching starts approximately 2 cm proximal from each end and continues distally.

A 100‐mm BioBrace (ConMed Linvatec, Largo, FL, USA) is looped through a 4.75 mm knotless suture anchor (Argo Knotless Suture Anchor, ConMed Linvatec, Largo, FL, USA) (Figure 3). Starting 2 cm proximal to the end, the folded BioBrace is then whipstitched. To do this, #2 looped suture tapes (Suture Loop Hi‐Fi Suture, ConMed Linvatec, Largo, FL, USA) are whipstitched, starting 2 cm proximal to the most distal end and continuing down to the most distal part of the BioBrace (Figure 4). If the BioBrace diameter is a concern, each end of the BioBrace can be whipstitched separately, allowing the 2 limbs to be passed one at a time through the distal portion of the ACL footprint (Figure 5).

### ACL Repair

**FIGURE 6:**
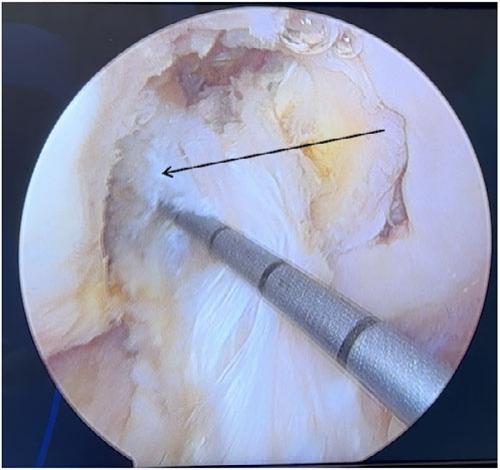
Shows a 30° arthroscopic view of the right knee. The anterior cruciate ligament can be seen torn from its posterolateral insertion (arrow).

**FIGURE 7:**
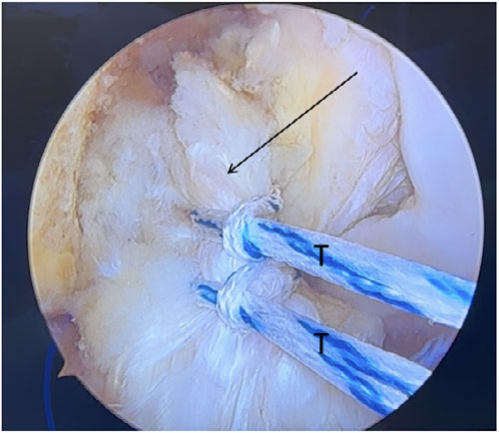
Shows a 30° arthroscopic view of the right knee joint. Two “luggage tag sutures” (T) have been cinched down onto the anterior cruciate ligament (arrow).

**FIGURE 8:**
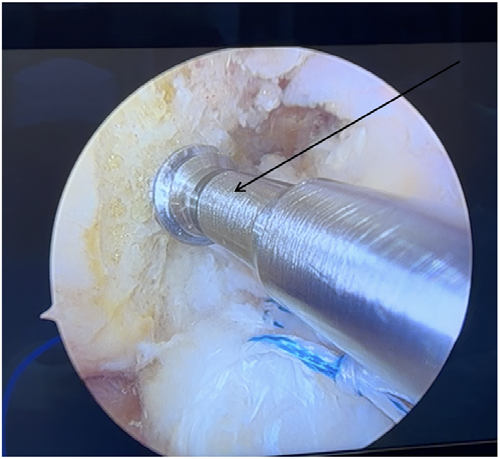
Shows a 30° arthroscopic view of the right knee. A hand tap (arrow) is placed into the notch.

**FIGURE 9:**
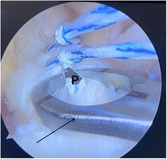
Shows a 30° arthroscopic view of the right knee. A tibial knee guide (arrow) is used to send a 2.4‐mm pin (P) through the anteromedial insertion of the ACL. (ACL, anterior cruciate ligament.)

The ACL shows laxity and a tear near the more proximal end of the ligament (Figure 6). A “luggage tag” suture (Suture Loop Hi‐Fi Suture, ConMed Linvatec, Largo, FL, USA) is passed through the anteromedial portal using a suture passer (Spectrum Auto Pass Suture Passer, ConMed, Largo, FL, USA) and is deployed into the proximal end of the ACL near the tear. The suture passer is removed, and the “luggage tag” is cinched down to secure the tissue. A second luggage tag is placed in a similar fashion (Figure 7). A 5.0 drill (ConMed Linvatec, Largo, FL, USA) is inserted into the lateral part of the intercondylar notch near the proximal attachment of the ACL, followed by a hand tap (ConMed Linvatec 4.75/5.50 mm Argo Knotless Punch, ConMed Linvatec, Largo, FL, USA) (Figure 8). Next, a tibial knee guide (Infinity Modular Guide System, ConMed Linvatec, Largo, FL, USA) is used to insert a 2.4‐ mm pin through the anteromedial insertion of the ACL, followed by a 3.5‐mm drill (Figure 9). A passing suture (Hi‐Fi Suture, ConMed, Largo, FL, USA) is placed through the tibial tunnel to maintain tunnel access for subsequent graft passage.

### BioBrace Augmentation

**FIGURE 10:**
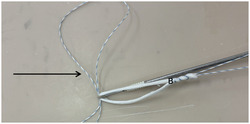
Shows the “luggage tag” suture (arrow) loaded into the knotless suture anchor with the BioBrace (B).

**FIGURE 11:**
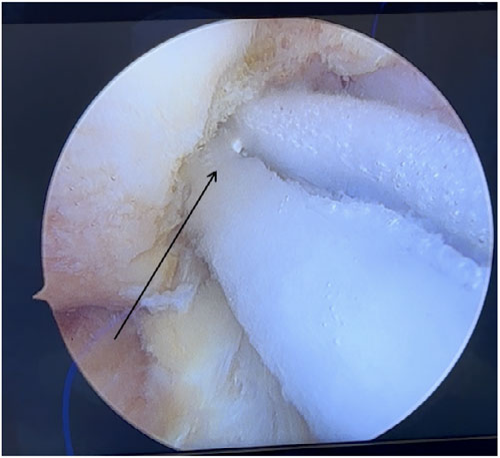
Shows a 30° arthroscopic view of the right knee. The BioBrace (arrow) is fixed into the lateral femoral condyle using the knotless suture anchor.

The BioBrace and the luggage tag sutures are loaded into the knotless suture anchor (Figure 10). The suture anchor is then inserted into the knee, and the BioBrace and luggage tag sutures are fixed into the predrilled part of the anatomic ACL insertion on the femoral notch (Figure 11). The suture anchor is then screwed in, placing tension on the sutures. The BioBrace is then pulled inferiorly toward the tibial tunnel through the anteromedial part of the ACL. If each end of the BioBrace has been whipstitched separately, the 2 limbs can be passed one at a time through the anteromedial portion of the ACL footprint.

### Fixation

**FIGURE 12:**
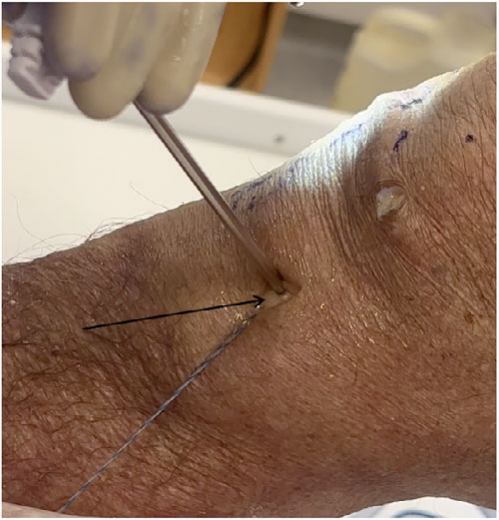
Shows tibial fixation of the right knee with a knotless suture anchor (arrow).

**FIGURE 13:**
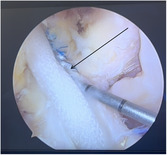
Shows a 30° arthroscopic view of the right knee. The BioBrace is shown, as well as the repaired anterior cruciate ligament (arrow).

Fixation on the tibial side is done using a 4.75‐mm knotless suture anchor (Argo Knotless Suture Anchor, ConMed Linvatec, Largo, FL, USA). A hand tap (ConMed Linvatec 4.75/5.50 mm Argo Knotless Punch, ConMed Linvatec, Largo, FL, USA) is used, followed by the knotless suture anchor (Figure 12). The repair is assessed arthroscopically to ensure proper fixation and tension (Figure 13).

## DISCUSSION

This technique describes a biocomposite scaffold implant to reinforce ACL repair. Early attempts at ACL repair showed high failure rates, with some studies illustrating failure rates up to 50%.^12^ These unfavorable outcomes are multifactorial, commonly associated with poor patient selection, age, and tear type.^13^ Augmentation with this scaffold offers protective features, including load‐sharing and enhanced ligament healing. Further, failure is largely attributed to the absence of protective augmentation to load‐share with the healing ligament and the lack of biologic augmentation to enhance the ACL's inherently poor healing environment.^8^, ^14^ Augmentation with this scaffold offers protective features, including load‐sharing and biological healing properties.

The BioBrace is a bovine collagen scaffold that has shown to integrate early with host tissue and facilitate the development of organized collagen fibers oriented along the axis of mechanical load in animal rotator cuff models.^15^ The BioBrace has also been used in human subjects in various sites for augmentation of both reconstructions and repairs of ligaments and tendons.^16^, ^17^, ^18^, ^19^ The BioBrace offers load‐sharing with approximately 280 N of tensile strength when doubled over (per ConMed, internal data) in addition to enhanced biological healing properties.^20^

There are several advantages of ACL repair compared with ACL reconstruction. Repair allows for preservation of the native ligament, which maintains proprioception and avoids the morbidity associated with autograft harvest.^21^, ^22^ This approach may enable quicker rehabilitation and return to activity.^23^ The current repair techniques that offer biological healing, such as the Bridge‐Enhanced ACL Repair implant, do not provide additional load‐sharing properties. However, the internal brace is recognized for its stabilizing and load‐sharing properties.^9^ This technique provides strength through load‐sharing, with studies showing repairs augmented with an internal brace have around 400 N higher load to failure than repairs alone, but it does not offer any additional biologic healing capabilities.^24^ The BioBrace remarkably combines properties found in both collagen scaffolds and suture tape augmentation, maintaining proprioceptive function and offering mechanical load‐sharing and biologic augmentation within one implant, thus promoting greater stability and healing.

While long‐term clinical outcome data for BioBrace‐augmented ACL repair are lacking, multiple ongoing studies assessing long‐term clinical outcomes are currently underway. Despite the absence of long‐term outcome data, BioBrace‐augmented ACL repair is an important technique to consider, as its benefits of combined load‐sharing and biologic properties offer notable reinforcement compared with alternative treatments.

## DISCLOSURES

The authors (W.S., A.M.) declare the following financial interests/ personal relationships which may be considered as potential competing interests: W.S. reports a relationship with Enovis that includes consulting or advisory. A.M. reports financial support was provided by ConMed; reports a relationship with ConMed that includes consulting or advisory; and serves on the Editorial Board of *Arthroscopy*. The other authors (C.B., E.P., J.B., T.E., K.W.) declare that they have no known competing financial interests or personal relationships that could have appeared to influence the work reported in this paper.

## FUNDING

This work was supported by the UAB Department of Orthopaedic Surgery.
